# A Novel Aryl Hydrocarbon Receptor Antagonist HBU651 Ameliorates Peripheral and Hypothalamic Inflammation in High-Fat Diet-Induced Obese Mice

**DOI:** 10.3390/ijms232314871

**Published:** 2022-11-28

**Authors:** Sora Kang, Aden Geonhee Lee, Suyeol Im, Seung Jun Oh, Hye Ji Yoon, Jeong Ho Park, Youngmi Kim Pak

**Affiliations:** 1 Department of Neuroscience, Graduate School, Kyung Hee University, Seoul 02447, Republic of Korea; 2 Department of Physiology, School of Medicine, Biomedical Science Institute, Kyung Hee University, Seoul 02447, Republic of Korea; 3 Phillips Exeter Academy, Exeter, NH 03833, USA; 4 Department of Biomedical Sciences, Graduate School, Kyung Hee University, Seoul 02447, Republic of Korea; 5 Department of Chemical & Biological Engineering, Hanbat National University, 125 Dongseodaero, Dukmyung-Dong, Yuseong-Gu, Daejeon 34158, Republic of Korea

**Keywords:** high-fat diet, inflammation, aryl hydrocarbon receptor (AhR), antagonist, hypothalamus

## Abstract

Obesity is a chronic peripheral inflammation condition that is strongly correlated with neurodegenerative diseases and associated with exposure to environmental chemicals. The aryl hydrocarbon receptor (AhR) is a ligand-activated nuclear receptor activated by environmental chemical, such as dioxins, and also is a regulator of inflammation through interacting with nuclear factor (NF)-κB. In this study, we evaluated the anti-obesity and anti-inflammatory activity of HBU651, a novel AhR antagonist. In BV2 microglia cells, HBU651 successfully inhibited lipopolysaccharide (LPS)-mediated nuclear localization of NF-κB and production of NF-κB-dependent proinflammatory cytokines, such as tumor necrosis factor (TNF)-α, interleukin (IL)-1β, and IL-6. It also restored LPS-induced mitochondrial dysfunction. While mice being fed a high-fat diet (HFD) induced peripheral and central inflammation and obesity, HBU651 alleviated HFD-induced obesity, insulin resistance, glucose intolerance, dyslipidemia, and liver enzyme activity, without hepatic and renal damage. HBU651 ameliorated the production of inflammatory cytokines and chemokines, proinflammatory Ly6c^high^ monocytes, and macrophage infiltration in the blood, liver, and adipose tissue. HBU651 also decreased microglial activation in the arcuate nucleus in the hypothalamus. These findings suggest that HBU651 may be a potential candidate for the treatment of obesity-related metabolic diseases.

## 1. Introduction

Neurodegenerative diseases involve the progressive structural and functional degeneration and death of neurons in specific regions of the central nervous system (CNS) [1]. Alzheimer’s disease (AD) and Parkinson’s disease (PD), and other neurodegenerative diseases, significantly impact the patient’s quality of life, causing memory loss, moodiness, anxiety, depression, and agitation, as well as loss of balance and movement, and affecting talking, breathing, and heart function [2,3].

Although the molecular mechanisms of neurodegenerative diseases have not been elucidated, numerous studies suggest that they are closely related to neuroinflammation [4]. Neuroinflammation is an inflammatory process in the CNS that is mediated by inflammatory and neurotoxic mediators, including tumor necrosis factor-alpha (TNF-α), interleukin-1β (IL-1β), IL-6, monocyte chemoattractant protein-1 (MCP-1/CCL2), reactive oxygen species (ROS), and nuclear factor kappa-B (NF-κB) [5,6,7]. In addition, inflammatory immune cells and inflammatory cytokines secreted from periphery organs can also cross the defective blood–brain barrier (BBB) and augment neuroinflammation [8,9].

Since obesity is a chronic low-grade inflammation condition [10], recent studies have suggested a strong correlation between obesity and the development of progressive aging-related neurodegenerative diseases [11]. Obesity induces hypoxic damage in adipose tissue, which produces proinflammatory cytokines such as TNF-α, IL-6, and IL-1β, as well as chemokines, such as MCP-1 [12]. Inflammatory cytokines disrupt the BBB, which allows chemokine-recruited monocytes to pass the BBB and change into inflammatory microglia [8]. Newly recruited microglia induce neuroinflammation, increase oxidative stress, and decrease mitochondria function, leading to neuronal cell death by apoptosis or necrosis, and also altering the synaptic plasticity of the neuron [13,14].

The aryl hydrocarbon receptor (AhR) is a ligand-activated nuclear receptor/transcription factor that is activated by various ligands, such as dioxin-like environmental chemicals [15], kynurenine tryptophan derivatives [16], dietary components [17], and low density lipoproteins [18]. AhR may be linked to obesity, because the ligand-activated AhR may disrupt fat metabolism and contribute to obesity [19]. Recent researches have shown that AhR plays an essential role in regulating chronic diseases [20] and inflammatory responses [21,22,23]. Therefore, modulation of abnormal AhR activation could be a promising approach to prevent obesity and ameliorate neurodegenerative diseases [24].

HBU651 is a novel, potent and selective AhR antagonist that may interact directly with the AhR ligand binding pocket and competes with an AhR ligand for binding to the AhR. In this study, we investigated the anti-obesity and anti-neuroinflammatory activity of HBU651 in lipopolysaccharide (LPS)-treated BV2 microglia cells and high-fat diet (HFD)-induced obesity mice.

## 2. Results

### 2.1. In Vitro Study

#### 2.1.1. HBU651 Is an AhR Antagonist

**Figure 1:**
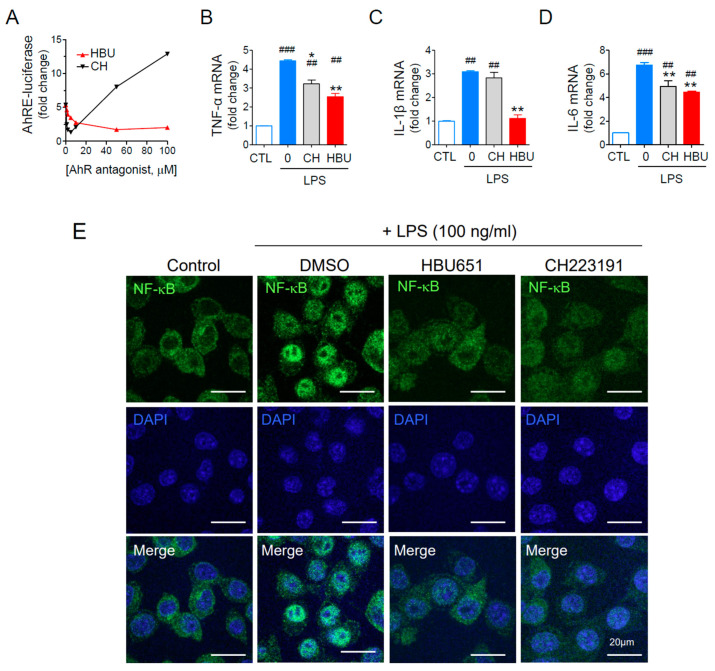
Effects of AhR antagonists on inflammatory cytokines and mitochondrial activities. (**A**) Dose-dependent AhR antagonist activity assay using AhR-dependent luciferase (AhRE-luciferase) assay. The cells were pretreated with AhR antagonists for 1 h, stimulated with 50 pM TCDD for 24 h, and assayed for luciferase activity. (**B**–**D**) Murine BV2 microglial cells were pre-treated with 10 μM HBU651 (HBU) or 1 μM CH223191 (CH) for 24 h, stimulated with 100 ng/mL LPS for 4 h, and harvested for analysis. Realtime qRT-PCR for TNF-α (**B**), IL-1β (**C**), and IL-6 (**D**) mRNA levels. Data are expressed as the mean ± SEM (*n* = 5). ^##^ *p* < 0.01, ^###^ *p* < 0.001 vs. Control (CTL), and * *p* < 0.05, ** *p* < 0.01 vs. LPS-treated DMSO. (**E**) Effect of HBU651 on LPS-induced nuclear localization of NF-κB. Murine BV2 microglial cells were pre-treated with 10 μM HBU651 or 1 μM CH223191 for 24 h, prior to 100 ng/mL LPS stimulation for 4 h. BV2 cells were immunostained with NF-κB p65/RELA antibody (green) and DAPI (blue) for nuclei (Merge). Cell images were enlarged to visualize the nuclear localization of NF-κB (Enlarged). Representative confocal micrograph images of BV2 cells are shown (scale bar = 20 μm).

To screen chemicals that inhibit AhR-mediated transcription activity, we used stable Hepa1c1c7 cells transfected with AhRE-luciferase reporter plasmid, as previously described [25,26]. The selected candidate chemical, HBU651, dose-dependently inhibited AhRE-luciferase activity activated with 50 pM 2,3,7,8-tetrachlorodibenzo-p-dioxin (TCDD) (Figure 1A). The half maximal inhibitory concentration (IC_50_) of HBU was approximately 10 μM. It should be noted that the reference AhR antagonst CH223191 (IC_50_ = 1 μM) [27] showed greater antagonistic activity at <5 μM, but the AhRE-luciferase activity was increased at >10 μM CH223191. Therefore, we used 10 μM HBU651 and 1 μM CH223191 for the subsequent in vitro experiments.

#### 2.1.2. Anti-Neuroinflammatory Effects of HBU651 on LPS-Stimulated BV2 Cells

The effect of HBU651 on LPS-induced neuroinflammation in BV2 cells was assessed by analyzing the mRNA levels of major proinflammatory cytokines such as TNF-α, IL-6, and IL-1β. LPS stimulation (100 ng/mL) increased these cytokine mRNA levels three- to sixfold in BV2 cells, whereas HBU651 treatment significantly reduced these levels (*p* < 0.01). HBU651 was more effective in reducing cytokines than the reference AhR antagonist CH223191 (Figure 1B–D).

NF-κB is a master transcription factor of inflammatory cytokines [28]. The transcription of proinflammatory cytokines and chemokines can be modified by AhR activation, interacting with NF-κB [29,30]. LPS stimulation induced phosphorylation and degradation of IκB and consequently induced nuclear localization of active NF-κB [31,32,33]. Immunocytochemical staining of NF-κB p65/RELA indicated that HB651 completely blocked the LPS-mediated nuclear localization of NF-κB p65/RELA (Figure 1E), suggesting that HBU651 can block the nuclear translocalization of NF-κB and NF-κB-dependent transcription of pro-inflammatory cytokines in LPS-activated microglial cells.

#### 2.1.3. The Effects of HBU651 on Mitochondrial Function in BV2 Cells

**Figure 2:**
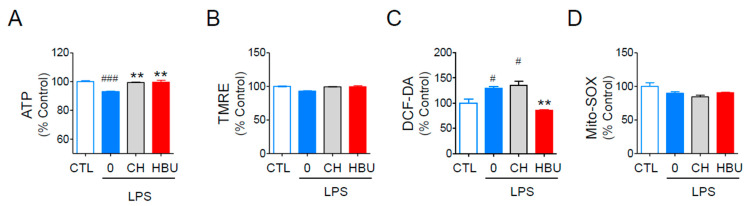
Effects of AhR antagonists on mitochondrial function. Murine BV2 microglial cells on a 96 well plate were pre-treated with 10 μM HBU651 (HBU) or 1 μM CH223191 (CH) for 24 h, stimulated with 100 ng/mL LPS for 4 h, and harvested for mitochondrial activity analysis. (**A**) intracellular ATP content, (**B**) TMRE-mediated mitochondrial membrane potential, and (**C**) DCF-DA-based total ROS generation. (**D**) Mito-Sox-based mitochondrial superoxide generation. Data are expressed as the mean ± SEM (*n* = 5). ^#^ *p* < 0.05, ^###^ *p* < 0.001 vs. Control (CTL), and ** *p* < 0.01 vs. LPS-treated DMSO.

LPS treatment decreased the mitochondrial activity, which could be monitored using the intracellular ATP contents and mitochondrial membrane potential, and ROS generation [34]. HBU651 significantly protected BV2 cells from LPS-induced decrease of intracellular ATP contents, at a level similar to CH223191 (Figure 2A). HBU651 also reduced LPS-mediated total cellular ROS production, as measured by staining with DCF-DA, but not CH223191 (Figure 2C). Neither HBU651 nor CH223191 altered TMRE-mitochondrial membrane potential and MitoSOX-superoxide production, as LPS did not significantly affect these values (Figure 2B,D).

### 2.2. In Vivo Study

#### 2.2.1. Metabolic Phenotypes

**Figure 3:**
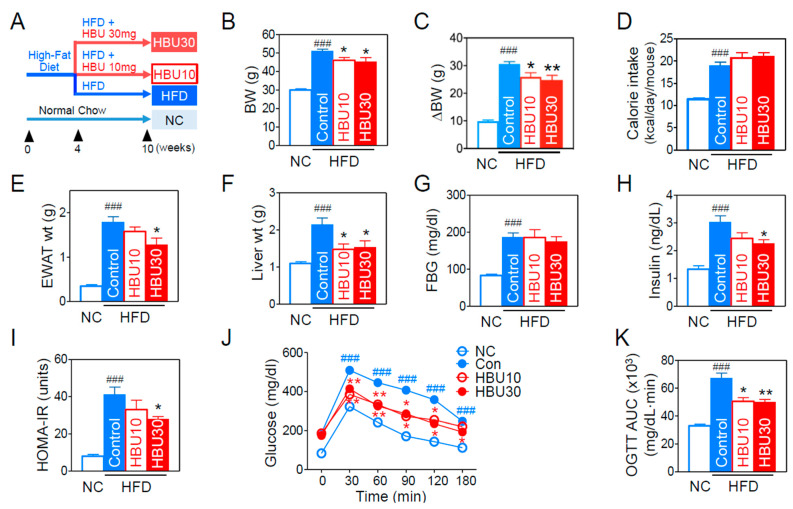
Effects of HBU651 on metabolic phenotypes in HFD-induced obese mice. (**A**) Experimental scheme in vivo, (**B**) Body weight (BW, g) at the end of the experiment, (**C**) Changes of BW, (**D**) Oral calories intake, (**E**) Epididymal white adipose tissue (EWAT) weight, (**F**) Liver weight, (**G**) Fasting blood glucose (FBG), (**H**) Fasting insulin, (**I**) Homeostatic model assessment of insulin resistance (HOMA-IR), (**J**) Time-course changes of blood glucose in the oral glucose tolerance test (OGTT), (**K**) Area under the curve (AUC) of OGTT. Data are expressed as means ± SEM (*n* = 5). ^###^ *p* < 0.001 vs. NC, and * *p* < 0.05, ** *p* < 0.01 vs. HFD-control. NC, normal chow; Control, HFD-control; HBU10, HFD plus HBU651 10 mg/kg; HBU30, HFD plus HBU651 30 mg/kg.

To investigate the anti-obesity effects of HBU651, mice were fed a HFD for 10 weeks with or without HBU651 oral administration, as summarized in Figure 3A. After 10 weeks of HFD, the HFD group had significantly gained body weight (BW, ΔBW) compared to the normal chow (NC) group (9.8 ± 0.4 g vs. 30.5 ± 0.9 g in ΔBW, *p* < 0.001, Figure 3B,C). The treatments with 10 mg/kg (HBU10) and 30 mg/kg (HBU30) HBU651 in HFD-fed mice significantly attenuated ΔBW (25.9 ± 1.4 g and 24.7 ± 1.7 g, respectively, *p* < 0.01, Figure 3C). Food intake and calorie intake were significantly increased compared to the NC group (*p* < 0.001), but the HBU10 and HBU30 groups showed no difference compared to the HFD control group (Figure 3D). Similarly to the body weight change, HFD also increased the weight of epididymal white adipose tissue (EWAT) and liver (*p* < 0.001 vs. NC), but HBU10 and HBU30 reduced these weights significantly compared to the HFD control (*p* < 0.05, Figure 3E,F). These results indicate that HBU651 alleviated the HFD-induced increase of weight gain in body, epididymal fat, and the liver, without affecting calorie intake.

#### 2.2.2. Glucose Metabolism and Insulin Resistance

The fasting blood glucose and fasting insulin levels in the HFD group were markedly elevated in comparison with the NC group (*p* < 0.001, Figure 3G,H). HBU30 treatment significantly improved fasting insulin (2.26 ± 0.13 vs. 3.09 ± 0.23 ng/dL, *p* < 0.05, Figure 3H), but did not improve fasting glucose compared to the HFD control (Figure 3G). Next, insulin resistance was assessed using homeostatic model assessment for insulin resistance (HOMA-IR) and with an oral glucose tolerance test (OGTT). HBU30 treatment also significantly alleviated HOMA-IR and OGTT compared to the HFD control (*p* < 0.05, Figure 3I–K). In OGTT, glucose levels at all time points were more elevated in the HFD group than in the NC group (Figure 3J). At 30, 60, 90, 120, and 180 min after glucose loading, glucose levels in the HBU10 and HBU30 groups were significantly reduced compared to the HFD control group (*p* < 0.01, Figure 3J). The area under the curve (AUC) of the HFD control group was significantly higher than the NC group, but HBU10 and HBU30 significantly decreased the AUC compared to the HFD control group (*p* < 0.01, Figure 3K).

#### 2.2.3. Lipid and Biochemical Parameters

**Figure 4:**
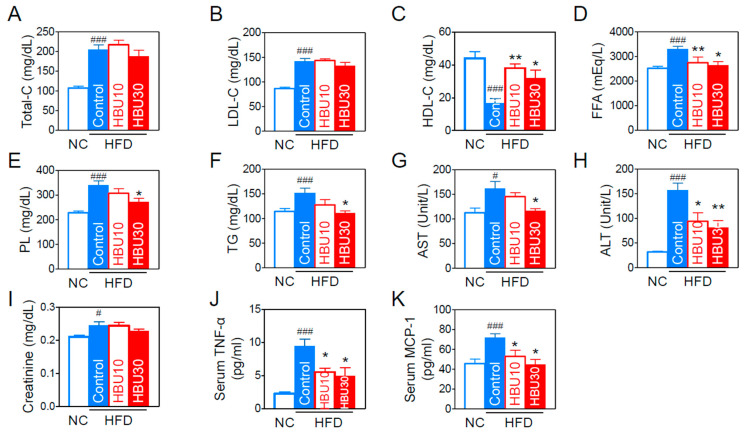
Effects of HBU651 on the lipid and biochemical profiles in the blood of HFD-induced obese mice. (**A**) Total-cholesterol (Total-C), (**B**) LDL-cholesterol (LDL-C), (**C**) HDL-cholesterol (HDL-C), (**D**) Free fatty acid (FFA), (**E**) Phospholipids (PL), (**F**) Triglyceride (TG), (**G**,**H**) Liver function marker enzyme activities of AST (**G**) and ALT (**H**), (**I**) Creatinine, (**J**,**K**) ELISA. Serum protein levels of TNF-α (**J**) and MCP-1 (**K**). Data are expressed as means ± SEM (*n* = 5). ^#^ *p* < 0.05, ^###^ *p* < 0.001 vs. NC, and * *p* < 0.05, ** *p* < 0.01 vs. HFD-control. NC, normal chow; Control, HFD-control; HBU10, HFD plus HBU651 10 mg/kg; HBU30, HFD plus HBU651 30 mg/kg.

As obesity influences the blood lipids and biochemical parameters, we analyzed the lipid profile and hepatic and renal biochemical parameters in the blood. HFD worsened all blood lipid profiles (Figure 4A–F), whereas HBU651 effectively improved the profiles of HDL-cholesterol (HDL-C, *p* < 0.05, Figure 4C), free fatty acid (FFA, *p* < 0.05, Figure 4D), phospholipid levels (PL, *p* < 0.05, Figure 4E), and triglyceride (TG, *p* < 0.05, Figure 4F). When measuring serum hepatic or renal injury markers for hepatic and renal toxicity, HBU651 treatment restored the HFD-induced increase in serum aspartate aminotransferase (AST, *p* < 0.05, Figure 4G) and alanine aminotransferase (ALT, *p* < 0.01, Figure 4H). However, HBU651 did not change the HFD-mediated increase in serum creatinine levels (Figure 4I). These results suggest that HBU651 administration did not induce any adverse toxic effects on the liver and kidney.

#### 2.2.4. Serum Inflammatory Cytokines and Chemokines

The pro-inflammatory cytokines and chemokines are strongly associated with obesity-induced peripheral and central inflammation. As a result of measuring serum TNF-α and MCP-1 levels using ELISA, the HFD increased serum TNF-α levels by 4.2-fold (*p* < 0.001) and MCP-1 by 1.6-fold (*p* < 0.001). HBU30 decreased HFD-induced TNF-α levels by approximately 48% of HFD control (*p* < 0.05) (Figure 4J) and normalized MCP-1 to the NC group (Figure 4K).

#### 2.2.5. Inflammatory Monocytes and Adipose Tissue Macrophages (ATMs)

**Figure 5:**
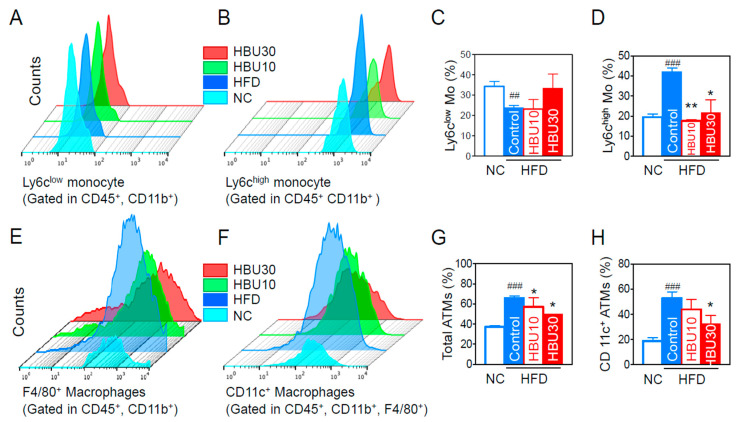
Effects of HBU651 on blood inflammatory monocytes and adipose tissue macrophages (ATM) in HFD-induced obese mice. (**A**) Flow cytometry of blood Ly6c^low^ monocytes (Mo), and (**B**) blood Ly6c^high^ monocytes, (**C**) Percentage of Ly6c^low^ monocytes, and (**D**) Ly6c^high^ monocytes, (**E**) Flow cytometry of CD45^+^, CD11b^+^, and F4/80^+^ ATM, and (**F**) CD11c^+^ inflammatory ATM, (**G**) Percentage of total ATMs, and (**H**) CD11c^+^ inflammatory ATM. Data are expressed as the mean ± SEM (*n* = 5). ^##^ *p* < 0.01, ^###^ *p* < 0.001 vs. NC, and * *p* < 0.05, ** *p* < 0.01 vs. HFD-control. NC, normal chow; Control, HFD-control; HBU10, HFD plus HBU651 10 mg/kg; HBU30, HFD plus HBU651 30 mg/kg.

Flow cytometry and flow-assisted cell sorting techniques permit immunophenotyping, quantification, and purification of monocytes and macrophages from blood or tissues. Monocytes secrete inflammatory cytokines and chemokines, and peripheral blood monocytes are subclassified into two populations (Ly6c^low^ and Ly6c^high^ monocytes) based on the expression of Ly6c. Under inflammatory conditions, Ly6c^high^ monocytes differentiate into M1 pro-inflammatory macrophages in the local cytokine environment [35]. We analyzed the population of Ly6c subtypes in CD45^+^/CD11b^+^ monocytes in peripheral blood. The HFD decreased the Ly6c^low^ monocytes (Figure 5A,C) and increased the proportion of Ly6c^high^ monocytes (*p* < 0.001, Figure 5B,D). HBU651 treatments decreased the Ly6c^high^ monocytes to NC levels (*p* < 0.01, Figure 5D), but did not significantly change the Ly6c^low^ monocytes (Figure 5C). However, the CD4^+^ T cell, CD8^+^ T cell, and CD4^+^/CD8^+^ ratios were not altered in all groups (data not shown).

Adipose tissue macrophages (ATMs) are the predominant source of secreted inflammatory cytokines [36]. We quantified ATMs and their subtype populations using flow cytometry. The proportion of total ATMs significantly increased in the HFD-control group (*p* < 0.001, Figure 5E,G). The proportion of total ATMs in the HBU651-treated groups was significantly lower than in the HFD-control group (*p* < 0.05, Figure 5G). Similarly, HFD increased the proportion of F4/80+/CD11c+ pro-inflammatory type 1 macrophages (M1), which differentiate from CD11b+/Ly6c^high^ monocytes and secrete TNF-α and MCP-1 (*p* < 0.001, Figure 5F,H). HBU651 administration decreased the proportion of F4/80+/CD11c+ M1 ATMs (*p* < 0.05, Figure 5H).

#### 2.2.6. Hepatic Steatosis and Cytokine mRNAs

**Figure 6:**
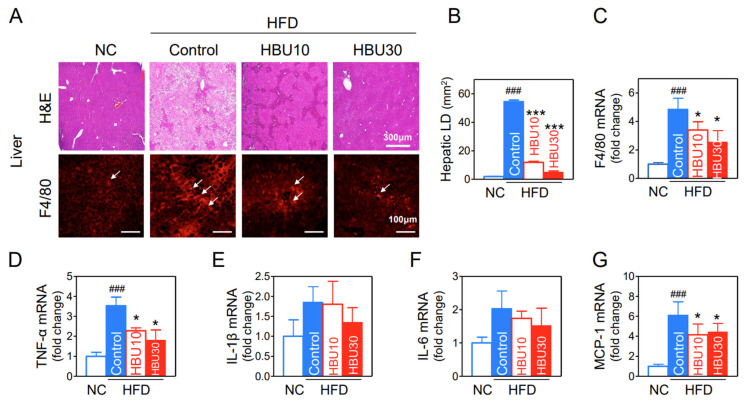
Effects of HBU651 on histological analysis and inflammatory gene expression of the liver in HFD-induced obese mice. (**A**) Liver H&E staining and F4/80 immunostaining, Arrows indicate F4/80 positive cells. (**B**) Hepatic lipid droplet (LD) area. (**C**–**G**) Realtime qRT-PCR of hepatic mRNA for F4/80 (**C**), TNF-α (**D**), IL-1β (**E**), IL-6 (**F**), and MCP-1 (**G**). Data are expressed as the mean ± SEM (*n* = 5). ^###^ *p* < 0.001 vs. NC, and * *p* < 0.05, *** *p* < 0.001 vs. HFD-control. NC, normal chow; Control, HFD-control; HBU10, HFD plus HBU651 10 mg/kg; HBU30, HFD plus HBU651 30 mg/kg.

Hepatic fat deposition results in insulin resistance, leading to increased levels of circulating inflammatory cytokines. The extent of fat accumulation in the liver of HFD mice was analyzed by measuring the area of hepatic lipid droplet (LD) in H&E stained liver (Figure 6A). HFD significantly increased the area of hepatic LDs (*p* < 0.001, Figure 6B), whereas HBU651 dramatically prevented fat accumulation in the liver, with respect to LD number and size (*p* < 0.001, Figure 6A,B). These fat accumulation results correlate with the liver weights in the study groups. Macrophage infiltration in HBU651-treated liver was analyzed using immunohistochemical staining, as excess fat deposition in the liver tissue expansion is the primary cause of macrophage recruitment. Hepatic F4/80-positive cells (red) were increased in the HFD control compared to the NC and decreased by HBU651 treatments (Figure 6A), consistent with the results for mRNA levels of F4/80 (*p* < 0.05, Figure 6C).

Real-time qRT-PCR for mRNA levels of cytokine (TNF-α, IL-6, IL-1β) and chemokine (MCP-1) in the liver showed that HFD upregulated the mRNA expression of TNF-α by 3.5-fold (*p* < 0.001) and MCP-1 by 6.1-fold (*p* < 0.001) in the liver (Figure 6D,G). The HBU651 treatment significantly reduced the mRNA levels of TNF-α (*p* < 0.05), MCP-1 (*p* < 0.05), and F4/80 (*p* < 0.05). Changes in IL-1β and IL-6 mRNA levels were similar to the other cytokines, but not statistically significant in all groups (Figure 6E,F).

#### 2.2.7. Fat Accumulation and Macrophage Infiltration in Adipose Tissue

**Figure 7:**
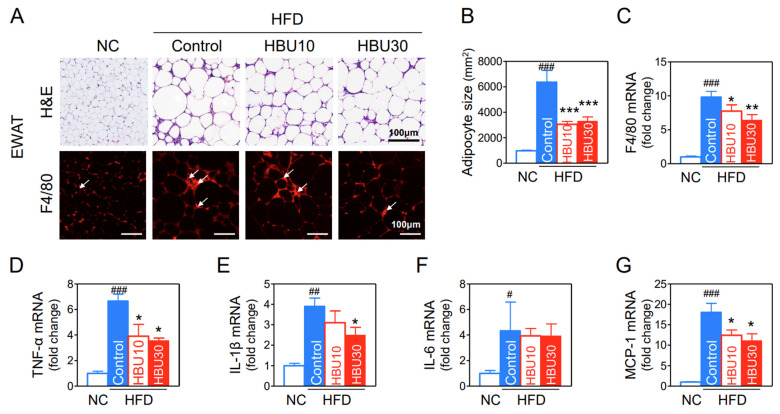
Effects of HBU651 on the histological analysis and inflammatory gene expression of the epididymal fat in HFD-induced obese mice. (**A**) EWAT H&E staining and F4/80 immunostaining, arrows indicate F4/80 positive cells. (**B**) Adipocyte size of the EWAT, (**C**–**G**) Realtime qRT-PCR of EWAT mRNA for F4/80 (**C**), TNF-α (**D**), IL-1β (**E**), IL-6 (**F**), and MCP-1 (**G**). Data are expressed as means ± SEM (*n* = 5). ^#^ *p* < 0.05, ^##^ *p* < 0.01, ^###^ *p* < 0.001 vs. NC, and * *p* < 0.05, ** *p* < 0.01, *** *p* < 0.001 vs. HFD-control. NC, normal chow; Control, HFD-control; HBU10, HFD plus HBU651 10 mg/kg; HBU30, HFD plus HBU651 30 mg/kg.

The size of adipocytes in the HFD group appeared to be markedly larger than those in the NC group (Figure 7A), whereas HBU651 treatment reduced the adipocyte size (*p* < 0.001, Figure 7B). Immunohistochemical staining of epididymal white adipose tissue (EWAT) for F4/80 exhibited a greater increase of macrophages in the HFD control group than in the NC group, and HBU651 treatment decreased F4/80-positive cells (Figure 7A).

In the gene expression of EWAT, the HFD enhanced F4/80 mRNA by 9.8-fold (*p* < 0.001), TNF-α mRNA by 6.6-fold (*p* < 0.001), IL-1β mRNA by 3.9-fold (*p* < 0.01), IL-6 mRNA by 4.3-fold (*p* < 0.05), and MCP-1 mRNA by 18.1-fold (*p* < 0.001) (Figure 7). HBU651 administration significantly reduced the mRNA levels of F4/80 (*p* < 0.01), TNF-α (*p* < 0.05), IL-1β (*p* < 0.05), and MCP-1 (*p* < 0.05) in EWAT (Figure 7). IL-6 mRNA levels were not altered by HBU651 in EWAT.

#### 2.2.8. Neuroinflammation in the Hypothalamus

HFD-induced inflammatory immune cells and cytokines in the peripheral liver and adipose tissue can cross the BBB and induce neuroinflammation in brain regions such as the hippocampus and the hypothalamus [8]. Inflammation of the arcuate nucleus (ARC) in the mediobasal hypothalamus develops rapidly, before obesity is established. Hypothalamic inflammation causes the changes in ARC neurons responsible for diet-induced obesity and insulin resistance.

**Figure 8:**
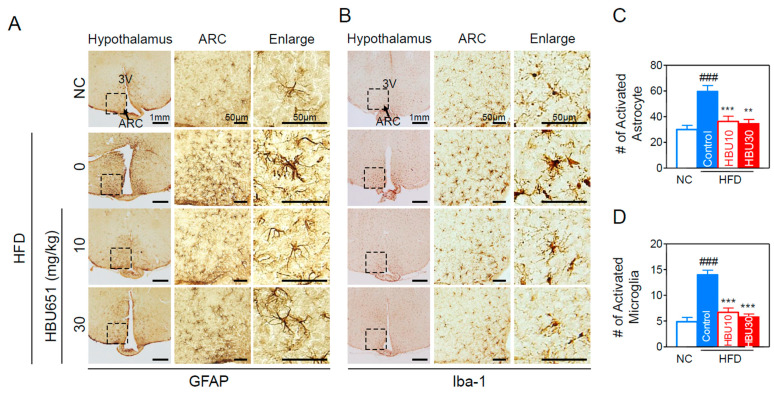
HBU651 attenuates HFD-induced hypothalamic inflammation. (**A**,**B**) Representative images and higher magnification inset of the hypothalamus of HFD-fed mice. Hypothalamic sections were immunostained with GFAP (**A**) or Iba-1 (**B**) antibodies. The arcuate nucleus (ARC) area (arrow, inset box) and the third ventricle (3V) are indicated. Box area in ARC has been enlarged to view the cellular morphology. (**C**,**D**) Quantification of astrocytes (GFAP-positive cells) (**C**) and activated microglia (Iba-1 positive cells) (**D**) in the ARC. Data are expressed as means ± SEM (*n =* 5). ^###^ *p* < 0.001 vs. NC, and ** *p* < 0.01. *** *p* < 0.001 vs. HFD-control. NC, normal chow; Control, HFD-control; HBU10, HFD plus HBU651 10 mg/kg; HBU30, HFD plus HBU651 30 mg/kg.

To check whether HBU651 treatment modulated hypothalamic inflammation, HFD-induced activation of microglia and astrocytes was determined using immunohistochemical staining of coronal sections of brain. HFD induced activation of GFAP-labeled astrocytes (Figure 8A) and Iba-1-labeled microglia (Figure 8B) in the ARC of the hypothalamus (inset box). HFD caused microglia accumulation in the ARC, with the Iba-1- or GFAP-labeled microglia displaying the typical morphology of activated cells with an enlarged soma size and amoeboid shape (Figure 8). HBU651 treatment decreased the number of activated GFAP-positive astrocytes (Figure 8A,C) and activated Iba1-positive microglia (Figure 8B,D).

## 3. Discussion

This is the first study to show that the novel AhR antagonist HBU651 exhibited anti-obesity and anti-inflammatory activities in BV2 microglial cells and HFD-induced obese mice. HBU651 inhibited the HFD-induced inflammatory responses in the liver, EWAT, and ARC of the hypothalamus, presumably by blocking the NF-κB signaling pathway that interacted with AhR. In the liver and EWAT, HBU651 significantly attenuated macrophage infiltration and the expression of proinflammatory cytokines and chemokines. More importantly, HBU651 decreased the number of activated microglia and astrocytes in the ARC of the hypothalamus.

AhR or dioxin receptor is a multifunctioning nuclear receptor in xenobiotic-induced toxicity and carcinogenesis, but also in some physiological functions such as reproduction, organ homeostasis, and adaptive immunity [37]. Upon AhR activation by xenobiotics, AhR translocates into the nucleus and dimerizes with the AhR nuclear translocator (Arnt). The AhR-Arnt heterodimer then binds to the AhR (or dioxin)-responsive element (AhRE/DRE) located upstream of target genes such as CYP1A1, CYP1B1, and AhRR [23]. Indeed, AhR is abundantly expressed in lung, liver [38], various brain regions [37,39], and barrier tissues, such as the skin, lungs, gut, and mucosa [40]. In human cohort studies, we reported that elevated serum concentrations of AhR agonist mixture were surrogate biomarkers for the future development of diabetes [25], insulin resistance [41], and renal failure [42]. Patients were exposed to a mixture of different environmental chemicals, including dioxins, dioxin-like polychlorinated biphenyls (PCBs), and bisphenol A, determined as mixtures of AhR agonist [25,43]. The concentration of AhR agonist mixture in the serum of diabetic patients was approximately 50–150 pM TCDD equivalent (TCDDeq), as assessed using an AhRE-luciferase assay [25]. In this study, based on these results, after stimulation with 50 pM TCDD, synthetic chemicals with AhR antagonistic activity were screened using the AhRE-luciferase assay system. The selected HBU651 was a specific AhR antagonist, without compromising cell viability or mitochondrial activity. To evaluate therapeutic efficacy on insulin resistance, we investigated the effect of HBU651 on inflammation using LPS-stimulated BV2 cells and an HFD-induced obese mice model.

The tryptophan metabolite kynurenine is an interesting endogenous AhR agonist that is elevated in chronic diseases such as cancers and neurological disorders [20,44]. The kynurenine pathway of tryptophan metabolism is altered in disease states, resulting in enhanced kynurenine levels. Kynurenine activated AhR pathway in brain tumors, cancer progression, and inflammation [44]. Meanwhile, AhR’s function is somewhat contradictory in the aging process: AhR activation promoted aging, whereas AhR expression decreased significantly with age [38]. Similarly, complex tissue and context-specific functions have been reported for AhR in immune response [45]. As AhR is highly expressed in a number of immune cells, AhR signaling plays important roles in the immune system in health and disease, and provides a molecular pathway to integrate the environment and metabolism in the immune responses [46]. In particular, ligand-activated AhR induces NF-κB-dependent transcription of inflammatory cytokines and chemokines [23,47], and may be linked with obesity and obesity-induced peripheral and central inflammation [48]. Various studies, including human cohort studies, support that inhibition of the AhR pathway or aberrant AhR activation may be a good therapeutic strategy for metabolic diseases. However, it should be noted that any attempt to modulate AhR function must take into consideration the dose, location, and timing of administration of AhR regulators, because AhR also plays important roles in normal cell physiology and function.

Insulin resistance is a pathophysiological condition caused by chronic peripheral inflammation [49,50], which is associated with metabolic syndrome, showing hyperglycemia, hyperinsulinemia, dyslipidemia, increased visceral adiposity, and hepatic steatosis [51]. In the lean state, insulin inhibits lipolysis and promotes glucose uptake in the essential insulin-sensitive tissues, skeletal muscle, liver, and adipose tissue [52]. However, in the obese state, impaired insulin sensitivity activates lipolysis to increase the concentration of circulating free fatty acids (FFAs). Increased FFA inhibits glucose uptake by muscle cells and increases hepatic glucose production [53], leading to ectopic lipid accumulation in both muscle and liver tissues [54]. Although the molecular mechanisms and mediators governing HFD-mediated insulin resistance and inflammation are unclear, one protein of interest in regulating the insulin response is AhR. Overexpression of AhR leads to insulin resistance, while AhR-null mice have enhanced insulin sensitivity and improved glucose tolerance [55]. Separately, it has been reported that HFD-induced exosome-derived phosphatidylcholine binds to and activates AhR, to inhibit the insulin signaling pathway and induce HFD-mediated insulin resistance [56].

In this study, HFD induced obesity-related symptoms, such as weight gain in the body, epididymal fat and liver, glucose intolerance (Figure 3), dyslipidemia, elevated liver enzymes (Figure 4), and fat accumulation of liver (Figure 6) and adipose tissue (Figure 7). HBU651 improved these symptoms and significantly reduced the FFAs, hepatic lipid droplets, and enzymes, to the level of the NC group. Therefore, considering that most of the glucose obtained from food intake is absorbed by the muscles and liver, and only 5% is taken by adipose tissue [57], and as HBU651 did not change fasting glucose (Figure 3G), the effects of HBU651 may be associated with an improvement of lipid and glucose utilization in the liver, rather than adipose tissue. The interaction of the AhR pathway with lipid and glucose metabolism needs to be studied in depth, to understand the molecular mechanisms.

Overnutrition-induced adipocyte hypertrophy causes hypoxic damage and stimulates the infiltration of Ly6c^high^ monocytes into various tissues by secreting MCP-1. Ly6c^high^ monocytes are more likely to differentiate into M1 inflammatory macrophages [58]. Macrophages are often classified into M1 classically-activated and M2 alternatively-activated macrophages [59]. M1 macrophages are characterized by the production of high levels of proinflammatory cytokines and ROS [60]. The newly recruited-inflammatory macrophages release several inflammatory cytokines, such as TNF-α, IL-1β, and IL-6, leading to insulin resistance in peripheral tissues, including adipose tissue, liver, and skeletal muscle. Transcription of inflammatory molecules is activated through the master inflammation transcription factor NF-κB-dependent pathway [28]. Thus, once these macrophages are activated and increased, they can develop into a vicious cycle involving macrophage recruitment and inflammatory cytokines production, consequently resulting in chronic peripheral inflammation. In BV2 microglial cells, HBU651-mediated AhR inactivation inhibited LPS-induced NF-κB activity and production of proinflammatory cytokines and ROS (Figure 1). In the blood, adipose tissue, and liver in obese mice, HBU651 decreased Ly6c^high^ monocytes, macrophages, and TNF-α and MCP-1 (Figure 5). The blockade of the NF-κB pathway by HBU651 may be the primary underlying mechanism for improving insulin resistance, glucose intolerance, and dyslipidemia.

Many clinical and animal studies have reported that the brain is also negatively affected by obesity. For example, the grey matter volumes in the temporal and frontal lobes are negatively correlated with body mass index [61]. The circulating inflammatory cytokines and immune cells released from peripheral tissues can cross the BBB and bind to its receptors at ARC in the mediobasal hypothalamus [62], resulting in hypothalamic inflammation. In addition, peripheral inflammation activates microglia and astrocytes, contributing to BBB disruption. Microglia, especially M1 microglia cells, may contribute to BBB dysfunction and vascular leakage through secretion of TNF-α, IL-1β, IL-6, IL-12, and MCP-1 [63]. Astrocytes also affect BBB permeability by producing proinflammatory mediators and oxidative stress, as well as promoting peripheral immune cell infiltration under an inflammatory condition [64]. The hypothalamus is directly or indirectly responsible for feeding and metabolism. Pathological inflammation in the hypothalamus is linked to the development and progression of obesity and its complications [35,65]. Furthermore, many of these functions are interrelated with the attention, learning, and memory aspects of cognition [66]. Thus, pharmacological or genetic inhibition of hypothalamic inflammation may lead to reduced food intake, increased insulin sensitivity, and peripheral inflammation.

In this study, HBU651 inhibited NF-κB activation, possibly by reducing AhR activity, in LPS-stimulated BV2 microglial cells, thereby inhibiting the production of TNF-α, IL-1β, IL-6, and ROS. HBU651 also restored mitochondrial dysfunction and ATP production. It was surprising that HBU651 treatment almost normalized the number of activated microglia and astrocytes in ARC of the hypothalamus, as well as all inflammatory phenotypes in the blood, liver, and adipose tissue. Combining the in vitro and in vivo results, AhR antagonist HBU651 may benefit chronic inflammatory diseases and peripheral inflammation, including hepatic steatosis and/or neuroinflammation, by blocking activation of macrophages, microglia, and astrocytes. HBU651 may modulate feeding and energy metabolism by regulating neuropeptides for appetite in the hypothalamus, consequently treating obesity and its complications, including neurodegenerative disease. We believe that HBU651-mediated inhibition of both peripheral and hypothalamic inflammation could facilitate the development of novel therapeutics to treat obesity-induced metabolic diseases.

## 4. Materials and Methods

### 4.1. Preparation of HBU651

HBU651 (2-(((1-(2-(1H-indol-3-yl)ethyl)-1H-1,2,3-triazol-4-yl)methyl)amino)naphthalene-1,4-dione) was chemically synthesized using click reaction of propargylated naphthalene with tryptamine azide, using a microwave reactor in Jung Ho Park’s Laboratory (Hanbat University, Daejeon, Republic of Korea). Propargylated naphthalene was synthesized from the reaction of 1,4-dihydroxy naphthalene and propargyl amine in the presence of cerium (III) chloride heptahydrate and triethylamine (TEA) in EtOH at room temperature for 48 h. HBU-651 was obtained as a brown solid (0.15 g, 79% yield), and its structure was verified by ^1^H NMR (400 MHz, DMSO-*d*_6_) and ^13^C NMR (100MHz, DMSO-*d*_6_).

### 4.2. AhR-Dependent Luciferase Reporter Assay

Hepa1c1c7 mouse hepatoma cells (CRL-2026) were cultured in α-minimum essential medium (MEM) supplemented with 10% FBS and 1% penicillin/streptomycin at 37 °C in 5% CO_2_ atmosphere. Hepa1c1c7 cells were co-transfected with pGL4-DRE-luc(puro+) and pRL-mTK, and puromycin (1 μg/mL)-resistant stable cells were selected as described previously [43]. To determine AhR-dependent luciferase activity (AhRE-luciferase), 70% confluent, stable cells in Dulbecco’s modified Eagle medium supplemented with 10% charcoal stripped-FBS were pre-treated with AhR antagonists (HBU651 or CH223191) for 1 h and stimulated with 50 pM 2,3,7,8-tetrachlorodibenzo-p-dioxin (TCDD) for 24 h. Luciferase activities were measured using a Dual-Glo Luciferase assay system (Promega, Madison, WI, USA) and luminometer (Berthold, Bad Wildbad, Germany) and subsequently normalized against *Renilla* luciferase activity. AhRE-luciferase activity is presented as a fold change over the AhRE-luciferase of the control.

### 4.3. Cell Culture and Treatment

BV2 murine microglia cells (ATCC, CRL-2469) were cultured in DMEM complete media. BV2 cells (2 × 10^5^ cells/well) were seeded in culture dishes (60 mm) for 24 h, followed by incubation in serum deficient DMEM media containing 0.5 % FBS for 16 h. Cells were pretreated with HBU651 (10 μg/mL) or CH223191 (1 μM, AhR antagonist, CAS #., 301326-22-7, Sigma-Aldrich, St. Louis, MO, USA) as a positive control for 24 h, followed by incubation with LPS (100 ng/mL) or DMSO vehicle for 4 h. The final concentration of DMSO was 0.1%.

### 4.4. Assays for Mitochondrial Function

A systemic cell-based mitochondrial function analysis system (CMAPS) was established using SH-SY5Y cells in 96 well plates [67]. This assay system covers quantitative assays for viability (Calcein), methyl thiazyl tetrazolium-mitochondrial dehydrogenase activity (MTT) assay for NADH dehydrogenase complex 1 activity, intracellular ATP content, TMRE-based mitochondrial membrane potential (TMRE), and the CM-H_2_DCFDA- or MitoSox-dependent reactive oxygen species (ROS) of cells. Functional changes to the mitochondria were evaluated using the CMAPS results.

### 4.5. Assays for NF-κB Activation

The effect of HBU651 on the nuclear translocation of the p65 subunit of NF-κB was examined using immunofluorescence assay, using confocal microscopy [34]. Briefly, BV2 cells were cultured on poly-L-lysine coated coverslips in 6 well plates, pretreated with 10 μM HBU651 or 1 μM CH223191 for 24 h, and stimulated with 100 ng/mL LPS for 4 h. The treated cells were fixed in 4% paraformaldehyde for 20 min at room temperature and then permeabilized with 0.1% Triton X-100 in PBS for 15 min and blocked with 3% BSA. The cells were sequentially incubated with polyclonal rabbit antibodies against anti-NF-κB p65/RELA (sc-372, Santa Cruz BioTech, Santa Cruz, CA, USA) at room temperature and FITC-conjugated goat anti-rabbit IgG at room temperature for 1 h. After washing with PBS, the samples were mounted with GEL/MOUNT (Biomeda, Foster City, CA, USA) and observed using a confocal fluorescence microscope (Carl Zeiss, Göttingen, Germany).

### 4.6. Real-Time Quantitative Reverse Transcription-PCR (qRT-PCR)

Total RNA from cultured cells or liver tissue was isolated using Trizol reagent (Invitrogen, Carlsbad, CA, USA). Total RNA from epididymal fat pads was extracted using a Mini RNA Isolation IITM (Zymo Research, Orange, CA, USA). Total RNA (2 μg) was reverse transcribed using MMLV reverse transcriptase (Promega, Madison, WI, USA) and 10 pM oligo dT primers (Invitrogen), according to each manufacturer’s instructions. The levels of mRNA were determined using real-time qRT-PCR using cytokine primers (TNF-α, IL-6, IL-1β), cDNA, and SYBR PCR master mix on Roter-Gene Q (Qiagen, Hilden, Germany) with 2x AmpiGene^®^ qPCR green Mix Lo-ROX (Enzo Biochem, New York, NY, USA) at 95 °C for 10 min, followed by 45 cycles of 95 °C for 5 s, 60 °C for 15 s, and 72 °C for 20 s. Sequences of primers include TNF-α (5′-AAGCCTGTAGCCCACGTCGTA-3′, and 5′-GGCACCACTAGTTGGTTGTCTTTG-3′), IL-6 (5′-AACGATGATGCACTTGCAGA-3′; 5′-GAGCATTGGAAATTGGGGTA-3′), IL-1β (5′-TAC GAG CCG TAG CCC AAA CA-3′; 5′-GATCGTAACGGAAGCGTGGA-3′), MCP-1 (5′-AGGTCCCTGTCATGCTTCTGG-3′; 5′-CTGCTGCTGGTGATCCTCTTG-3′), F4/80 (5′-CTTTGGCTATGGGCTTCCAGTC-3′; 5′-GCAAGGAGGACAGAGTTTATCGTG-3′), 18S rRNA (5′-GAG CGA AAG CAT TTG CCA. AG-3′; 5′-GGC ATC GTT TAT GGT CGG AA-3′). Quantification of mRNA expression was analyzed using SDS Software 2.4 (Applied Biosystems^®^, Waltham, MA, USA). Each replicate cycle threshold (Ct) was normalized to 18S. The comparative Ct method was used to calculate the relative quantification of gene expression. The difference of Ct (ΔCt) means the difference between the 18S and the gene of interest. The fold change was normalized to the control condition, which was set as 1.

### 4.7. Experimental Scheme of High Fat Diet (HFD)-Fed Mice

Six-week-old male C57BL/6 mice weighing 19–21g from Central Lab Animals, Inc. (Seoul, Republic of Korea) were obtained in this study. The animals were housed in controlled lighting conditions (12 h light, 12 h night) and humidity (40–70%). Water and food were supplied ad libitum. After a week of adaptation, mice were given normal chow (NC, Research Diets D12450B, 10% kcal fat) or a high-fat diet (HFD, Research Diets D12492, 60% kcal fat). After 4 weeks of NC or HFD feeding, mice were assigned into four groups, according to diet and treatments: NC group (*n* = 5), HFD group (*n* = 5), HFD plus 10 mg/kg HBU651 group (HBU10, *n* = 5), HFD plus 30 mg/kg HBU651 group (HBU30, *n* = 5). The HBU10 and HBU30 groups were orally administered HBU651 10 mg/kg and 30 mg/kg once a day for 6 weeks, respectively, while the NC and HFD groups received saline. Animal maintenance and treatment were carried out following the Principles of Laboratory Animal Care (NIH publication No. 85–23, revised 1985) and the Animal Care and Use guidelines of Kyung Hee University, Seoul, Republic of Korea. The Animal Research Ethics Committee of Kyung Hee University, Seoul, Republic of Korea, approved this study (KHSASP-20-163). At week 10, we sacrificed the mice, measured the weight of tissues, and stored them at –70 °C until use.

### 4.8. Metabolic Phenotype Measurements

The body weight (BW) of each mouse was recorded during the experiment using an electronic scale (CAS 2.5D, Seoul, Republic of Korea). BW was measured at the same time before morning feeding. Food intake was calculated by averaging the daily food intake per mouse, by subtracting the remaining feed weight before feeding the next day from the previous day’s feed per cage at the dietary change every morning. Calorie intake was calculated by multiplying 2.91 kcal/g in the NC group and 5.24 kcal/g in the HFD group.

At 9 weeks, oral glucose tolerance test (OGTT) and homeostatic model assessment for insulin resistance (HOMA-IR) were performed after mice were fasted for 14 h in a clean cage. Before OGTT, the blood of mice was withdrawn from the tail vein to measure the levels of fasting glucose and fasting insulin. For OGTT, glucose (2 g/kg body weight) dissolved in distilled water was orally administered to each mouse. Blood were taken from the lateral tail vein at 0, 30, 60, 90, 120, and 180 min after oral glucose administration. The glucose level was measured using a strip-operated blood glucose meter (ACCU-CHEK Performa, Seoul, Republic of Korea), and insulin levels were analyzed using an enzyme-linked immunosorbent assay (Crystal Chem, Elk Grove Village, IL, USA). HOMA-IR was calculated according to the formula: HOMA-IR = (fasting glucose (mg/dL) × fasting blood insulin (ng/mL))/22.5.

### 4.9. Biochemical Analysis

At week 10, blood was collected from the heart under anesthesia. Clinical parameters, including total cholesterol (Total-C), low-density lipoprotein cholesterol (LDL-C), high-density lipoprotein cholesterol (HDL-C), phospholipid (PL), triglyceride (TG), and free fatty acid (FFA), were measured. Aspartate aminotransferase (AST), alanine aminotransferase (ALT), and creatinine were measured for liver and kidney function [68].

### 4.10. Serum TNF-α and MCP-1 Protein Levels

The serum concentration of inflammatory cytokine TNF-α, and chemokine MCP-1, were measured using a mouse TNF-α and mouse MCP-1 ELISA kit (BMS607-3 and BMS6005, Invitrogen, Carlsbad, CA, USA), according to the manufacturer’s protocol.

### 4.11. Preparation of Stromal Vascular Cells (SVCs)

Stromal vascular cells (SVCs) were isolated from epididymal fat pads using a well-established collagenase-based method, as previously described [35]. Briefly, mouse adipose tissue samples were isolated from the epididymal fat pad at 10 weeks. The samples in PBS buffer containing 2% BSA were cut into small pieces of 1–2 mm in size, and digested with collagenase type 2 (10 mg/mL, Worthington) and deoxyribonuclease I (2 mg/mL, Roche, Indianapolis, IN, USA) at 37 °C for 30 min with shaking. The filtered tissue lysates were centrifuged at 1000 rpm forv3 min. The pellets containing SVCs were resuspended with 2% FBS/PBS and prepared for staining.

### 4.12. Flow Cytometric Analysis of Blood Immune Cells and Adipose Tissue Macrophages (ATMs)

At 9 weeks, blood samples were collected from the tail veins of mice. After applying EDTA, we counted the number of blood cells using a Cellometer (Nexcelom Bioscience LLC, MA, USA) and adjusted it to 10^5^ cells for each sample. Then, 1% Fc Block (BD Biosciences, CA, USA) was added to each sample at the ratio of 1:100 and reacted for 10 min, and then stained with fluorophore-conjugated antibodies in the dark for 20 min. Antibodies used for blood lymphocytes and Ly6c monocytes analysis were CD45-APC Cyanine7, CD11b-phycoerythrin Cyanine7, CD3-FITC, CD4-PerCP CY5.5, CD8-phycoerythrin, and Ly6c-APC. Isolated SVCs were incubated with 1% Fc Block (BD Biosciences) and then stained with CD45-APC Cyanine7, CD11b-phycoerythrin Cyanine7, F4/80-APC, and CD11c-phycoerythrin. All antibodies were purchased from BioLegend (San Diego, CA, USA). After washing with 2% FBS/PBS solution and centrifuging at 1500 rpm, we transferred samples into a FACS tube and analyzed them using flow cytometry, BD Canto (BD Biosciences). To analyze the blood Ly6c monocytes, Ly6c^+^ and CD11b^+^ cells were analyzed after CD45+ gating; for blood lymphocytes, CD3^+^ T cells, CD4^+^ T cells, and CD8^+^ T cells were analyzed after CD45^+^ gating. Adipose tissue macrophages (ATMs) were identified as the F4/80^+^ ATMs (total ATM) and CD11c^+^ M1 ATMs after CD45^+^, CD11b^+^, and F4/80^+^ gating. The number of the immune cells was analyzed using the FlowJo FACS analysis program (Tree Star, Inc., Ashland, OR, USA).

### 4.13. Histological Analysis of the Liver and Epididymal Fat Pad

Liver and epididymal fat pads were fixed in a 4% paraformaldehyde (PFA) solution and created paraffin blocks. We cut each block into 5 μm thick slices using a microtome and placed each on a slide. For hematoxylin and eosin (H&E) and immunohistochemistry (IHC) staining, the slides of two tissues per animal were deparaffinized with xylene and gradient rehydrated. The images were captured with a computer-connected BX50 microscope (Olympus Optical, Tokyo, Japan). Finally, we measured the adipocyte size of the epididymal fat pad and the lipid droplet in the liver with ImageJ.

### 4.14. Brain Tissue Preparation and Immunohistochemical Staining

Whole brains were isolated from the skull, postfixed overnight with 4% paraformaldehyde in 0.1 M phosphate buffer (PB) at 4 °C, and then stored in 30% sucrose solution in 0.05 M PBS at 4 °C, until they sank. The brains were frozen-sectioned on a Cryostat (Microsystems AG, Leica, Wetzlar, Germany) with 30 μm thick coronal sections and stored in a cryoprotectant (25% ethylene glycol, 25% glycerol, 0.2 M PB, and water) at 4 °C until stained. All sections were collected in six different series and processed for immunostaining, as previously described [31]. Brain coronal sections (30 μm in thickness) containing the hypothalamus were incubated with rabbit anti-glial fibrillary acidic protein (anti-GFAP, 1:5000; Neuromics, Edina, MN, USA) or rabbit anti-ionized calcium binding adaptor molecule 1 (anti-Iba-1, 1:1000; Wako, Osaka, Japan), followed by staining with biotinylated anti-rabbit IgG and an avidin-biotin-peroxidase complex (ABC) standard kit (Vector Laboratories, Burlingame, CA, USA). Signals were detected by incubating sections with 0.5 mg/mL 3,3′-diaminobenzidine (Sigma, St. Louis, MO, USA) in 0.1 M PBS containing 0.003% H_2_O_2_.

To quantify the resting and activated microglia and astrocytes in the hypothalamus, coronal brain sections (30 μm thickness) were collected (5 sections/series), labeled with anti-Iba-1 or anti-GFAP antibodies, and imaged under a bright-field microscope (Olympus Optical, Tokyo, Japan). The sections labeled with Iba-1- or GFAP were digitized and manually counted within preselected fields (500 × 400 μm) of the hypothalamus (2 fields/animal). Activated microglia and astrocytes were classified and counted according to their morphologies, as previously described [35].

### 4.15. Statistical Analysis

All statistical analyses were performed using InStat (GraphPad Software, San Diego, CA, USA). One-way analysis of variance (ANOVA) was used to determine the levels of difference between groups, followed by a Tuckey’s post hoc test. All experimental data are presented as means ± standard error of the mean (SEM). All *p*-values were two-tailed, and *p* < 0.05 was considered statistically significant.

## 5. Conclusions

The novel AhR antagonist HBU651 exhibited anti-obesity and anti-inflammatory activities in vitro and in vivo. HBU651 inhibited HFD-induced inflammatory responses in the liver, EWAT, and ARC of the hypothalamus. Blockade of the NF-κB signaling pathway that interacts with AhR is proposed as the underlying mechanism for the decrease in the peripheral and central proinflammatory cytokines and chemokines. These findings suggest that HBU651 may be a potential multi-target therapeutic candidate for obesity-induced chronic inflammatory and metabolic diseases. Further studies, including clinical trials, are needed to confirm these results.

## 6. Patents

Patent application for HBU651 has been submitted.
